# 2,15-Dioxa-7,18,19,20,23-penta­aza­hepta­cyclo­[21.6.1.1^17,20^.0^1,8^.0^3,7^.0^9,14^.0^24,29^]hentriaconta-9,11,13,17(31),18,24,26,28-octaen-30-one

**DOI:** 10.1107/S1600536813027396

**Published:** 2013-10-12

**Authors:** Govindarajulu Rangabashyam Subhashree, Santhanagopalan Purushothaman, Raghavachary Raghunathan, Devadasan Velmurugan, Dasararaju Gayathri

**Affiliations:** a Department of Physics, Dr. M.G.R. Educational and Research Institute University, Periyar E.V.R. High Road, Adayalampattu, Chennai 600 095, India; b Department of Organic Chemistry, University of Madras, Guindy Campus, Chennai 600 025, India; c Centre of Advanced Study in Crystallography and Biophysics, University of Madras, Guindy Campus, Chennai 600 025, India; d Department of Biotechnology, Dr. M.G.R. Educational and Research Institute University, Periyar E.V.R. High Road, Maduravoyal, Chennai 600 095, India

## Abstract

In the title compound, C_24_H_23_N_5_O_3_, the oxindole ring system is nearly planar, with a dihedral angle between the two fused rings of 3.3 (1)°. In the fused pyrrolo–oxazole ring system, the oxazole and pyrrolidine rings adopt envelope conformations with the spiro C atom and one of the methyl­ene C atoms, respectively, as the flap atoms. In the crystal, mol­ecules are linked into a helical chain along the *b* axis *via* C—H⋯O inter­actions generating *R*_2_^1^(7) and *R*_2_^2^(8) ring motifs.

## Related literature

For the biological activity of pyrrole, oxazole and indole derivatives, see: Fernandes *et al.* (2004 ▶); Jiang *et al.* (2004 ▶). For a related crystal structure, see: Narayanan *et al.* (2013 ▶).
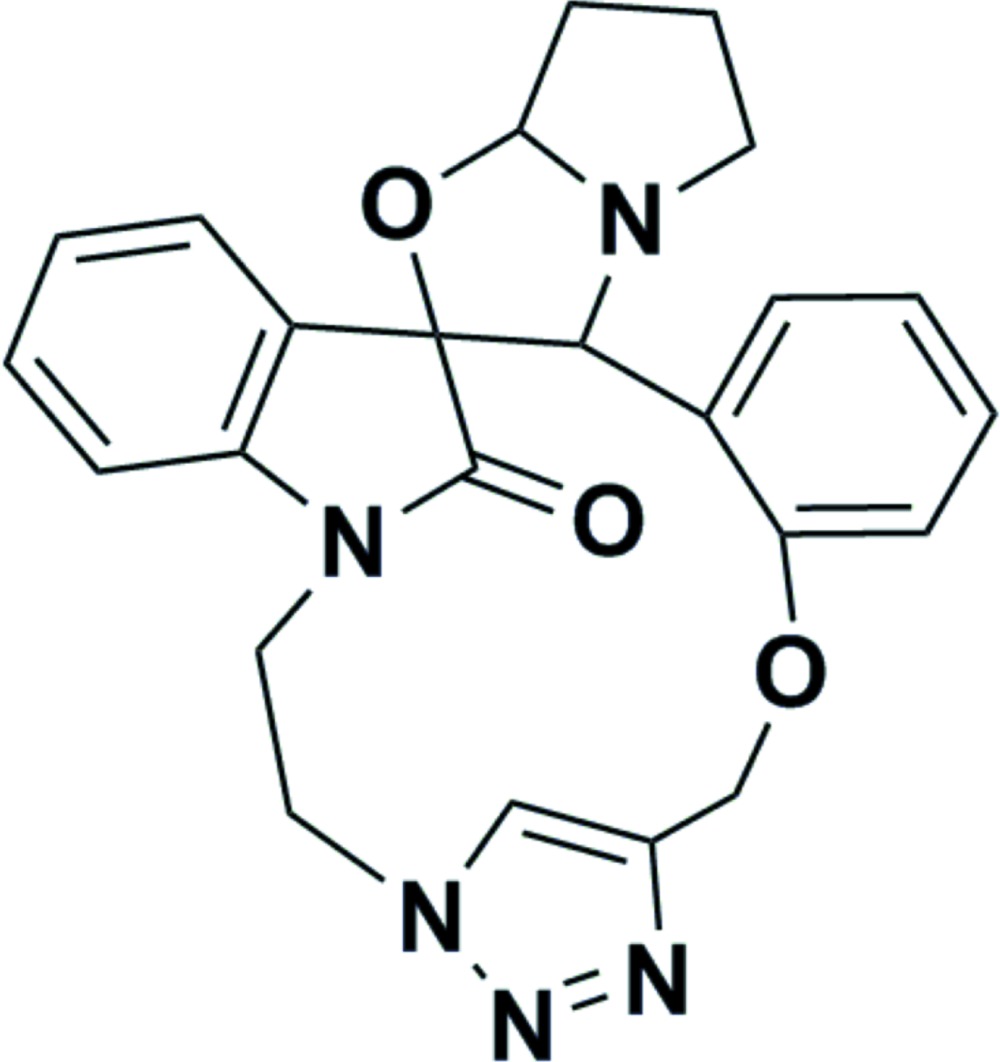


## Experimental

### 

#### Crystal data

C_24_H_23_N_5_O_3_
*M* *_r_* = 429.47
Monoclinic, 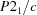

*a* = 9.273 (5) Å
*b* = 10.983 (5) Å
*c* = 20.096 (5) Å
β = 90.038 (5)°
*V* = 2046.7 (15) Å^3^
*Z* = 4
Mo *K*α radiation
μ = 0.10 mm^−1^
*T* = 293 K
0.30 × 0.25 × 0.20 mm

#### Data collection

Bruker SMART APEXII area-detector diffractometer
Absorption correction: multi-scan (*SADABS*; Bruker, 2008 ▶) *T*_min_ = 0.653, *T*_max_ = 0.746
18864 measured reflections
5113 independent reflections
3845 reflections with *I* > 2σ(*I*)
*R*_int_ = 0.027

#### Refinement

*R*[*F* ^2^ > 2σ(*F* ^2^)] = 0.050
*wR*(*F* ^2^) = 0.146
*S* = 1.01
5113 reflections
289 parameters
H-atom parameters constrained
Δρ_max_ = 0.56 e Å^−3^
Δρ_min_ = −0.25 e Å^−3^

### 

Data collection: *APEX2* (Bruker, 2008 ▶); cell refinement: *SAINT* (Bruker, 2008 ▶); data reduction: *SAINT*; program(s) used to solve structure: *SHELXS97* (Sheldrick, 2008 ▶); program(s) used to refine structure: *SHELXL97* (Sheldrick, 2008 ▶); molecular graphics: *ORTEP-3 for Windows* (Farrugia, 2012 ▶); software used to prepare material for publication: *SHELXL97* and *PLATON* (Spek, 2009 ▶).

## Supplementary Material

Crystal structure: contains datablock(s) I, global. DOI: 10.1107/S1600536813027396/is5309sup1.cif

Structure factors: contains datablock(s) I. DOI: 10.1107/S1600536813027396/is5309Isup2.hkl

Supplementary material file. DOI: 10.1107/S1600536813027396/is5309Isup3.cml

Additional supplementary materials:  crystallographic information; 3D view; checkCIF report

## Figures and Tables

**Table 1:** Hydrogen-bond geometry (Å, °)

*D*—H⋯*A*	*D*—H	H⋯*A*	*D*⋯*A*	*D*—H⋯*A*
C3—H3⋯O1^i^	0.93	2.45	3.311 (3)	154
C23—H23*B*⋯O2^i^	0.97	2.53	3.372 (3)	145
C24—H24*B*⋯O1^i^	0.97	2.31	3.209 (3)	153

Symmetry code: (i) 
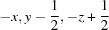
.

**Fig. 1.**
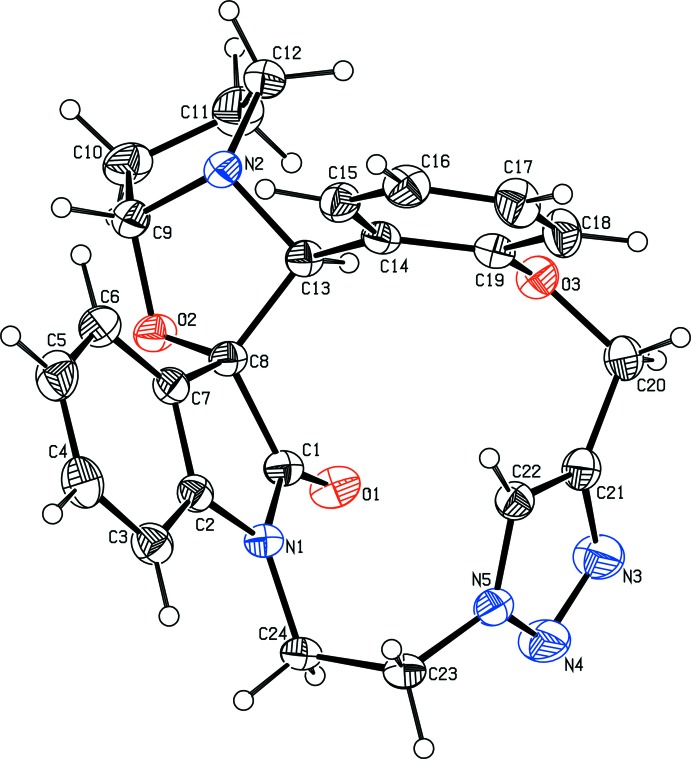
The molecular structure of the title compound, showing 30% probability displacement ellipsoids.

**Fig. 2.**
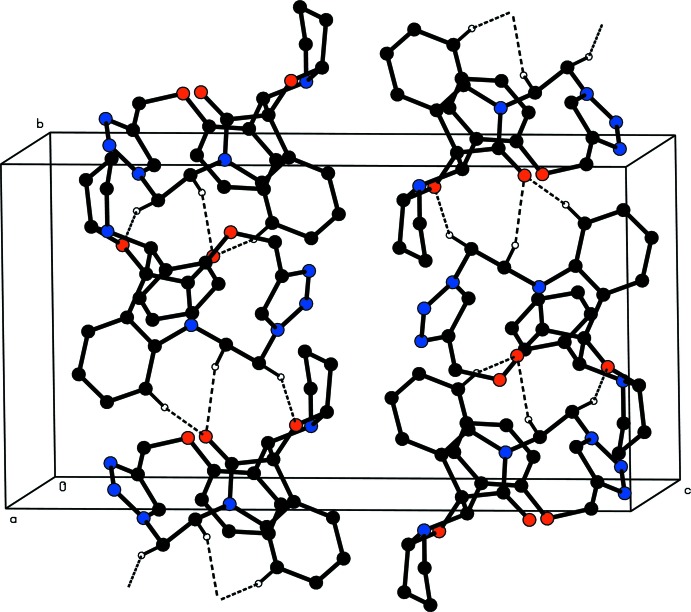
The molecular packing of the title compound. For clarity, hydrogen atoms which are not involved in hydrogen bonding are omitted.

## supplementary crystallographic information

### 1. Comment

Macrocylces play a major role in drug development for their remarkable biological activities. Pyrrole, oxazole and indole derivatives possess various pharmaceutical properties (Fernandes *et al.*, 2004; Jiang *et al.*, 2004). In view of their potential bioactivity, we have synthesized series of macrocycles and report here the crystal structure of the title compound.

The bond lengths and bond angles are within the normal ranges and are comparable with the similar crystal structure (Narayanan *et al.*, 2013). Traizole ring is planar and the oxindole moiety is nearly planar with a dihedral angle between the five- and six-membered rings being 3.3 (1)°. Atom O1 deviates by -0.080 (1) Å from the plane of the five-membered ring in oxindole moiety. In the pyrrolo-oxazole ring system, both pyrrolidine and oxazole rings adopt envelope conformations with C8 and C11 atoms deviating by -0.649 (2) and 0.565 (3) Å, respectively, from the mean plane of rest of the atoms in the corresponding rings. The crystal packing is stabilized by C—H···O intermolecular interactions, C3—H3···O1, C23—H23B···O2 and C24—H24B···O1 (Table 1), generating C(6), C(7) & C(5) chains, respectively, running along [010]. The intermolecular interaction network also generates *R*_2_^1^(7), *R*_2_^2^(8) and *R*_2_^2^(11) ring motifs.

### 2. Experimental

Solution of (2-((1-(2-(2,3-dioxoindolin-1-yl)ethyl)-1*H*-1,2,3-triazol-4-yl) methoxy)benzaldehyde (300 mg, 0.79 mmol) and *L*-proline (90 mg, 0.79 mmol) was refluxed in dry toluene (50 ml) under N2 atmosphere for 4 h under Dean–Stark apparatus. After the completion of reaction as indicated by TLC, toluene was evaporated under reduced pressure. The crude product was washed with water and extracted with dichloromethane (4×20 ml). The combined organic layers were dried (MgSO_4_), filtered and concentrated in vacuum. The crude product was purified by column chromatography using hexane: EtOAc (6:4) as eluent.

### 3. Refinement

All H-atoms were refined using a riding model with C—H = 0.93, 0.98 and 0.97 Å for aromatic, methine and methylene groups, respectively, and with *U*_iso_(H) = 1.2*U*_eq_(C).

### Crystal data

**Table:**

C_24_H_23_N_5_O_3_	*F*(000) = 904
*M**_r_* = 429.47	*D*_x_ = 1.394 Mg m^−^^3^
Monoclinic, *P*2_1_/*c*	Mo *K*α radiation, λ = 0.71073 Å
Hall symbol: -P 2ybc	Cell parameters from 5113 reflections
*a* = 9.273 (5) Å	θ = 2.0–28.4°
*b* = 10.983 (5) Å	µ = 0.10 mm^−^^1^
*c* = 20.096 (5) Å	*T* = 293 K
β = 90.038 (5)°	Block, colourless
*V* = 2046.7 (15) Å^3^	0.30 × 0.25 × 0.20 mm
*Z* = 4	

### Data collection

**Table:**

Bruker SMART APEXII area-detector diffractometer	5113 independent reflections
Radiation source: fine-focus sealed tube	3845 reflections with *I* > 2σ(*I*)
Graphite monochromator	*R*_int_ = 0.027
ω and φ scans	θ_max_ = 28.4°, θ_min_ = 2.0°
Absorption correction: multi-scan (*SADABS*; Bruker, 2008)	*h* = −12→12
*T*_min_ = 0.653, *T*_max_ = 0.746	*k* = −12→14
18864 measured reflections	*l* = −25→26

### Refinement

**Table:**

Refinement on *F*^2^	Primary atom site location: structure-invariant direct methods
Least-squares matrix: full	Secondary atom site location: difference Fourier map
*R*[*F*^2^ > 2σ(*F*^2^)] = 0.050	Hydrogen site location: inferred from neighbouring sites
*wR*(*F*^2^) = 0.146	H-atom parameters constrained
*S* = 1.01	*w* = 1/[σ^2^(*F*_o_^2^) + (0.0709*P*)^2^ + 0.8032*P*] where *P* = (*F*_o_^2^ + 2*F*_c_^2^)/3
5113 reflections	(Δ/σ)_max_ < 0.001
289 parameters	Δρ_max_ = 0.56 e Å^−^^3^
0 restraints	Δρ_min_ = −0.25 e Å^−^^3^

### Special details

**Table:**

Geometry. All e.s.d.'s (except the e.s.d. in the dihedral angle between two l.s. planes) are estimated using the full covariance matrix. The cell e.s.d.'s are taken into account individually in the estimation of e.s.d.'s in distances, angles and torsion angles; correlations between e.s.d.'s in cell parameters are only used when they are defined by crystal symmetry. An approximate (isotropic) treatment of cell e.s.d.'s is used for estimating e.s.d.'s involving l.s. planes.
Refinement. Refinement of *F*^2^ against ALL reflections. The weighted *R*-factor *wR* and goodness of fit *S* are based on *F*^2^, conventional *R*-factors *R* are based on *F*, with *F* set to zero for negative *F*^2^. The threshold expression of *F*^2^ > σ(*F*^2^) is used only for calculating *R*-factors(gt) *etc*. and is not relevant to the choice of reflections for refinement. *R*-factors based on *F*^2^ are statistically about twice as large as those based on *F*, and *R*- factors based on ALL data will be even larger.

### Fractional atomic coordinates and isotropic or equivalent isotropic displacement parameters (Å^2^)

**Table:**

	*x*	*y*	*z*	*U*_iso_*/*U*_eq_	
C1	0.14019 (16)	0.05291 (14)	0.29426 (8)	0.0343 (3)	
C2	0.12581 (16)	−0.13095 (15)	0.34750 (8)	0.0345 (3)	
C3	0.09293 (19)	−0.25119 (17)	0.36051 (10)	0.0439 (4)	
H3	0.0563	−0.3021	0.3276	0.053*	
C4	0.1166 (2)	−0.29295 (19)	0.42455 (11)	0.0523 (5)	
H4	0.0961	−0.3737	0.4348	0.063*	
C5	0.1700 (2)	−0.2177 (2)	0.47336 (10)	0.0551 (5)	
H5	0.1858	−0.2483	0.5159	0.066*	
C6	0.2005 (2)	−0.09606 (19)	0.45972 (9)	0.0481 (4)	
H6	0.2342	−0.0447	0.4931	0.058*	
C7	0.17987 (16)	−0.05276 (15)	0.39565 (8)	0.0358 (3)	
C8	0.19783 (16)	0.07191 (14)	0.36545 (8)	0.0339 (3)	
C9	0.18801 (18)	0.20933 (17)	0.45133 (9)	0.0414 (4)	
H9	0.1564	0.1690	0.4923	0.050*	
C10	0.1736 (2)	0.34643 (19)	0.45780 (11)	0.0542 (5)	
H10A	0.0888	0.3754	0.4344	0.065*	
H10B	0.1667	0.3702	0.5042	0.065*	
C11	0.3081 (3)	0.3960 (2)	0.42698 (13)	0.0631 (6)	
H11A	0.3340	0.4741	0.4462	0.076*	
H11B	0.2976	0.4049	0.3792	0.076*	
C12	0.4193 (2)	0.29847 (19)	0.44428 (10)	0.0508 (5)	
H12A	0.5002	0.3015	0.4138	0.061*	
H12B	0.4549	0.3095	0.4893	0.061*	
C13	0.34670 (17)	0.13335 (15)	0.36914 (8)	0.0353 (3)	
H13	0.3473	0.2022	0.3381	0.042*	
C14	0.48023 (16)	0.05760 (15)	0.35651 (8)	0.0346 (3)	
C15	0.52497 (18)	−0.02832 (17)	0.40269 (9)	0.0420 (4)	
H15	0.4738	−0.0365	0.4422	0.050*	
C16	0.64310 (19)	−0.10212 (19)	0.39183 (10)	0.0480 (4)	
H16	0.6693	−0.1604	0.4232	0.058*	
C17	0.7216 (2)	−0.0889 (2)	0.33447 (11)	0.0544 (5)	
H17	0.8000	−0.1396	0.3264	0.065*	
C18	0.6847 (2)	−0.0009 (2)	0.28880 (10)	0.0512 (5)	
H18	0.7405	0.0099	0.2508	0.061*	
C19	0.56425 (17)	0.07204 (15)	0.29935 (8)	0.0371 (4)	
C20	0.5354 (2)	0.13510 (18)	0.18557 (9)	0.0469 (4)	
H20A	0.6291	0.1004	0.1756	0.056*	
H20B	0.5249	0.2093	0.1598	0.056*	
C21	0.4204 (2)	0.04717 (17)	0.16601 (9)	0.0425 (4)	
C22	0.37870 (18)	−0.05874 (16)	0.19571 (8)	0.0382 (4)	
H22	0.4218	−0.0976	0.2317	0.046*	
C23	0.15348 (19)	−0.18146 (16)	0.18336 (9)	0.0405 (4)	
H23A	0.1993	−0.2465	0.2083	0.049*	
H23B	0.1060	−0.2170	0.1450	0.049*	
C24	0.04305 (17)	−0.11776 (16)	0.22683 (8)	0.0384 (4)	
H24A	−0.0017	−0.0527	0.2015	0.046*	
H24B	−0.0318	−0.1754	0.2388	0.046*	
N1	0.10500 (14)	−0.06718 (12)	0.28709 (7)	0.0343 (3)	
N2	0.34184 (15)	0.18234 (14)	0.43800 (7)	0.0392 (3)	
N3	0.3288 (2)	0.07077 (18)	0.11524 (9)	0.0630 (5)	
N4	0.2310 (2)	−0.01524 (18)	0.11278 (9)	0.0621 (5)	
N5	0.26167 (16)	−0.09446 (14)	0.16155 (7)	0.0408 (3)	
O1	0.12691 (15)	0.13061 (11)	0.25218 (6)	0.0473 (3)	
O2	0.10994 (12)	0.16351 (11)	0.39546 (6)	0.0419 (3)	
O3	0.52789 (14)	0.16379 (11)	0.25560 (6)	0.0445 (3)	

### Atomic displacement parameters (Å^2^)

**Table:**

	*U*^11^	*U*^22^	*U*^33^	*U*^12^	*U*^13^	*U*^23^
C1	0.0311 (7)	0.0327 (8)	0.0390 (8)	0.0058 (6)	−0.0041 (6)	−0.0042 (6)
C2	0.0289 (7)	0.0377 (8)	0.0369 (8)	0.0034 (6)	0.0002 (6)	0.0002 (6)
C3	0.0418 (9)	0.0390 (9)	0.0510 (10)	−0.0013 (7)	0.0015 (7)	−0.0013 (8)
C4	0.0520 (10)	0.0443 (10)	0.0607 (12)	0.0003 (8)	0.0091 (9)	0.0121 (9)
C5	0.0568 (11)	0.0630 (13)	0.0456 (11)	0.0005 (10)	0.0025 (9)	0.0183 (9)
C6	0.0483 (10)	0.0574 (11)	0.0385 (9)	−0.0030 (8)	−0.0035 (8)	0.0037 (8)
C7	0.0308 (7)	0.0399 (9)	0.0366 (8)	0.0009 (6)	−0.0002 (6)	−0.0002 (7)
C8	0.0319 (7)	0.0343 (8)	0.0356 (8)	0.0041 (6)	−0.0028 (6)	−0.0055 (6)
C9	0.0382 (8)	0.0478 (10)	0.0382 (9)	−0.0004 (7)	0.0018 (7)	−0.0065 (7)
C10	0.0558 (11)	0.0492 (11)	0.0576 (12)	0.0085 (9)	−0.0027 (9)	−0.0204 (9)
C11	0.0768 (15)	0.0423 (11)	0.0701 (14)	−0.0039 (10)	0.0060 (11)	−0.0097 (10)
C12	0.0473 (10)	0.0571 (12)	0.0481 (10)	−0.0134 (9)	0.0030 (8)	−0.0109 (9)
C13	0.0351 (8)	0.0342 (8)	0.0364 (8)	0.0017 (6)	−0.0016 (6)	−0.0018 (6)
C14	0.0293 (7)	0.0356 (8)	0.0390 (8)	−0.0007 (6)	−0.0051 (6)	−0.0050 (6)
C15	0.0394 (8)	0.0490 (10)	0.0377 (9)	0.0038 (7)	−0.0033 (7)	0.0000 (7)
C16	0.0417 (9)	0.0525 (11)	0.0497 (11)	0.0107 (8)	−0.0111 (8)	0.0049 (8)
C17	0.0377 (9)	0.0632 (13)	0.0624 (12)	0.0178 (9)	−0.0008 (8)	0.0031 (10)
C18	0.0396 (9)	0.0613 (12)	0.0527 (11)	0.0057 (8)	0.0088 (8)	0.0031 (9)
C19	0.0344 (8)	0.0353 (8)	0.0416 (9)	−0.0025 (6)	−0.0060 (6)	−0.0004 (7)
C20	0.0514 (10)	0.0455 (10)	0.0437 (10)	−0.0083 (8)	0.0048 (8)	0.0087 (8)
C21	0.0461 (9)	0.0436 (9)	0.0379 (9)	−0.0018 (7)	0.0018 (7)	0.0051 (7)
C22	0.0375 (8)	0.0390 (9)	0.0382 (9)	0.0014 (7)	−0.0012 (7)	0.0005 (7)
C23	0.0431 (9)	0.0373 (9)	0.0411 (9)	−0.0044 (7)	−0.0049 (7)	−0.0062 (7)
C24	0.0351 (8)	0.0410 (9)	0.0390 (9)	−0.0038 (7)	−0.0076 (7)	−0.0040 (7)
N1	0.0353 (6)	0.0328 (7)	0.0346 (7)	−0.0012 (5)	−0.0045 (5)	−0.0035 (5)
N2	0.0377 (7)	0.0445 (8)	0.0355 (7)	−0.0011 (6)	0.0009 (6)	−0.0070 (6)
N3	0.0723 (12)	0.0685 (12)	0.0481 (10)	−0.0187 (10)	−0.0146 (8)	0.0199 (9)
N4	0.0712 (11)	0.0696 (12)	0.0454 (9)	−0.0163 (10)	−0.0184 (8)	0.0174 (9)
N5	0.0442 (8)	0.0435 (8)	0.0348 (7)	−0.0030 (6)	−0.0020 (6)	−0.0017 (6)
O1	0.0613 (8)	0.0359 (6)	0.0447 (7)	0.0082 (6)	−0.0128 (6)	0.0028 (5)
O2	0.0372 (6)	0.0436 (7)	0.0448 (7)	0.0082 (5)	−0.0033 (5)	−0.0134 (5)
O3	0.0525 (7)	0.0382 (6)	0.0429 (7)	−0.0017 (5)	0.0005 (5)	0.0035 (5)

### Geometric parameters (Å, º)

**Table:**

C1—O1	1.208 (2)	C13—C14	1.513 (2)
C1—N1	1.366 (2)	C13—H13	0.9800
C1—C8	1.541 (2)	C14—C15	1.387 (2)
C2—C3	1.380 (3)	C14—C19	1.397 (2)
C2—C7	1.387 (2)	C15—C16	1.380 (3)
C2—N1	1.415 (2)	C15—H15	0.9300
C3—C4	1.384 (3)	C16—C17	1.371 (3)
C3—H3	0.9300	C16—H16	0.9300
C4—C5	1.375 (3)	C17—C18	1.376 (3)
C4—H4	0.9300	C17—H17	0.9300
C5—C6	1.393 (3)	C18—C19	1.391 (3)
C5—H5	0.9300	C18—H18	0.9300
C6—C7	1.386 (2)	C19—O3	1.379 (2)
C6—H6	0.9300	C20—O3	1.444 (2)
C7—C8	1.507 (2)	C20—C21	1.491 (3)
C8—O2	1.4285 (19)	C20—H20A	0.9700
C8—C13	1.538 (2)	C20—H20B	0.9700
C9—O2	1.427 (2)	C21—N3	1.352 (2)
C9—N2	1.482 (2)	C21—C22	1.363 (2)
C9—C10	1.517 (3)	C22—N5	1.342 (2)
C9—H9	0.9800	C22—H22	0.9300
C10—C11	1.496 (3)	C23—N5	1.453 (2)
C10—H10A	0.9700	C23—C24	1.517 (2)
C10—H10B	0.9700	C23—H23A	0.9700
C11—C12	1.527 (3)	C23—H23B	0.9700
C11—H11A	0.9700	C24—N1	1.451 (2)
C11—H11B	0.9700	C24—H24A	0.9700
C12—N2	1.469 (2)	C24—H24B	0.9700
C12—H12A	0.9700	N3—N4	1.310 (3)
C12—H12B	0.9700	N4—N5	1.341 (2)
C13—N2	1.485 (2)		
			
O1—C1—N1	125.74 (15)	C8—C13—H13	108.2
O1—C1—C8	126.13 (15)	C15—C14—C19	117.42 (15)
N1—C1—C8	108.13 (13)	C15—C14—C13	120.41 (15)
C3—C2—C7	122.67 (16)	C19—C14—C13	122.16 (15)
C3—C2—N1	127.32 (16)	C16—C15—C14	122.07 (17)
C7—C2—N1	109.94 (14)	C16—C15—H15	119.0
C2—C3—C4	117.28 (18)	C14—C15—H15	119.0
C2—C3—H3	121.4	C17—C16—C15	119.49 (18)
C4—C3—H3	121.4	C17—C16—H16	120.3
C5—C4—C3	121.42 (19)	C15—C16—H16	120.3
C5—C4—H4	119.3	C16—C17—C18	120.24 (17)
C3—C4—H4	119.3	C16—C17—H17	119.9
C4—C5—C6	120.62 (18)	C18—C17—H17	119.9
C4—C5—H5	119.7	C17—C18—C19	120.12 (17)
C6—C5—H5	119.7	C17—C18—H18	119.9
C7—C6—C5	118.95 (18)	C19—C18—H18	119.9
C7—C6—H6	120.5	O3—C19—C18	121.30 (16)
C5—C6—H6	120.5	O3—C19—C14	118.09 (15)
C2—C7—C6	119.03 (16)	C18—C19—C14	120.55 (16)
C2—C7—C8	108.75 (14)	O3—C20—C21	111.30 (14)
C6—C7—C8	132.13 (16)	O3—C20—H20A	109.4
O2—C8—C7	113.99 (13)	C21—C20—H20A	109.4
O2—C8—C13	100.54 (12)	O3—C20—H20B	109.4
C7—C8—C13	118.59 (13)	C21—C20—H20B	109.4
O2—C8—C1	106.84 (12)	H20A—C20—H20B	108.0
C7—C8—C1	102.27 (13)	N3—C21—C22	108.41 (17)
C13—C8—C1	114.51 (13)	N3—C21—C20	121.57 (17)
O2—C9—N2	105.97 (13)	C22—C21—C20	129.78 (17)
O2—C9—C10	111.89 (16)	N5—C22—C21	104.75 (15)
N2—C9—C10	107.38 (15)	N5—C22—H22	127.6
O2—C9—H9	110.5	C21—C22—H22	127.6
N2—C9—H9	110.5	N5—C23—C24	109.67 (14)
C10—C9—H9	110.5	N5—C23—H23A	109.7
C11—C10—C9	104.64 (16)	C24—C23—H23A	109.7
C11—C10—H10A	110.8	N5—C23—H23B	109.7
C9—C10—H10A	110.8	C24—C23—H23B	109.7
C11—C10—H10B	110.8	H23A—C23—H23B	108.2
C9—C10—H10B	110.8	N1—C24—C23	112.96 (13)
H10A—C10—H10B	108.9	N1—C24—H24A	109.0
C10—C11—C12	102.31 (18)	C23—C24—H24A	109.0
C10—C11—H11A	111.3	N1—C24—H24B	109.0
C12—C11—H11A	111.3	C23—C24—H24B	109.0
C10—C11—H11B	111.3	H24A—C24—H24B	107.8
C12—C11—H11B	111.3	C1—N1—C2	110.80 (13)
H11A—C11—H11B	109.2	C1—N1—C24	123.51 (14)
N2—C12—C11	105.04 (16)	C2—N1—C24	125.49 (14)
N2—C12—H12A	110.7	C12—N2—C9	106.36 (14)
C11—C12—H12A	110.7	C12—N2—C13	112.30 (14)
N2—C12—H12B	110.7	C9—N2—C13	105.71 (12)
C11—C12—H12B	110.7	N4—N3—C21	108.94 (16)
H12A—C12—H12B	108.8	N3—N4—N5	107.09 (15)
N2—C13—C14	112.38 (13)	N4—N5—C22	110.80 (15)
N2—C13—C8	100.14 (12)	N4—N5—C23	120.06 (15)
C14—C13—C8	119.02 (14)	C22—N5—C23	126.58 (15)
N2—C13—H13	108.2	C9—O2—C8	106.93 (12)
C14—C13—H13	108.2	C19—O3—C20	116.75 (14)
			
C7—C2—C3—C4	−0.3 (2)	C13—C14—C19—O3	4.1 (2)
N1—C2—C3—C4	−177.07 (16)	C15—C14—C19—C18	2.3 (2)
C2—C3—C4—C5	0.4 (3)	C13—C14—C19—C18	−178.74 (16)
C3—C4—C5—C6	0.6 (3)	O3—C20—C21—N3	126.6 (2)
C4—C5—C6—C7	−1.7 (3)	O3—C20—C21—C22	−47.2 (3)
C3—C2—C7—C6	−0.7 (2)	N3—C21—C22—N5	−0.5 (2)
N1—C2—C7—C6	176.53 (15)	C20—C21—C22—N5	173.87 (18)
C3—C2—C7—C8	−177.75 (15)	N5—C23—C24—N1	−62.19 (19)
N1—C2—C7—C8	−0.49 (17)	O1—C1—N1—C2	−176.26 (15)
C5—C6—C7—C2	1.7 (3)	C8—C1—N1—C2	3.07 (17)
C5—C6—C7—C8	177.89 (17)	O1—C1—N1—C24	−1.2 (3)
C2—C7—C8—O2	117.07 (14)	C8—C1—N1—C24	178.13 (13)
C6—C7—C8—O2	−59.4 (2)	C3—C2—N1—C1	175.41 (16)
C2—C7—C8—C13	−124.86 (15)	C7—C2—N1—C1	−1.69 (18)
C6—C7—C8—C13	58.7 (2)	C3—C2—N1—C24	0.5 (3)
C2—C7—C8—C1	2.16 (16)	C7—C2—N1—C24	−176.63 (14)
C6—C7—C8—C1	−174.33 (17)	C23—C24—N1—C1	99.69 (18)
O1—C1—C8—O2	56.1 (2)	C23—C24—N1—C2	−85.98 (19)
N1—C1—C8—O2	−123.19 (14)	C11—C12—N2—C9	−27.44 (19)
O1—C1—C8—C7	176.18 (16)	C11—C12—N2—C13	87.73 (18)
N1—C1—C8—C7	−3.15 (16)	O2—C9—N2—C12	126.13 (15)
O1—C1—C8—C13	−54.2 (2)	C10—C9—N2—C12	6.41 (19)
N1—C1—C8—C13	126.45 (14)	O2—C9—N2—C13	6.58 (18)
O2—C9—C10—C11	−98.40 (19)	C10—C9—N2—C13	−113.15 (16)
N2—C9—C10—C11	17.5 (2)	C14—C13—N2—C12	86.45 (18)
C9—C10—C11—C12	−33.3 (2)	C8—C13—N2—C12	−146.21 (14)
C10—C11—C12—N2	38.0 (2)	C14—C13—N2—C9	−157.99 (14)
O2—C8—C13—N2	43.82 (14)	C8—C13—N2—C9	−30.64 (16)
C7—C8—C13—N2	−81.10 (16)	C22—C21—N3—N4	0.8 (2)
C1—C8—C13—N2	157.94 (13)	C20—C21—N3—N4	−174.12 (19)
O2—C8—C13—C14	166.61 (14)	C21—N3—N4—N5	−0.8 (3)
C7—C8—C13—C14	41.7 (2)	N3—N4—N5—C22	0.5 (2)
C1—C8—C13—C14	−79.27 (18)	N3—N4—N5—C23	163.50 (17)
N2—C13—C14—C15	45.4 (2)	C21—C22—N5—N4	0.0 (2)
C8—C13—C14—C15	−71.2 (2)	C21—C22—N5—C23	−161.64 (16)
N2—C13—C14—C19	−133.54 (16)	C24—C23—N5—N4	−76.5 (2)
C8—C13—C14—C19	109.96 (18)	C24—C23—N5—C22	83.6 (2)
C19—C14—C15—C16	−3.4 (3)	N2—C9—O2—C8	22.94 (18)
C13—C14—C15—C16	177.61 (16)	C10—C9—O2—C8	139.67 (15)
C14—C15—C16—C17	1.6 (3)	C7—C8—O2—C9	86.14 (16)
C15—C16—C17—C18	1.5 (3)	C13—C8—O2—C9	−41.85 (16)
C16—C17—C18—C19	−2.5 (3)	C1—C8—O2—C9	−161.66 (13)
C17—C18—C19—O3	177.63 (18)	C18—C19—O3—C20	44.2 (2)
C17—C18—C19—C14	0.6 (3)	C14—C19—O3—C20	−138.69 (16)
C15—C14—C19—O3	−174.82 (14)	C21—C20—O3—C19	69.3 (2)

### Hydrogen-bond geometry (Å, º)

**Table:**

*D*—H···*A*	*D*—H	H···*A*	*D*···*A*	*D*—H···*A*
C3—H3···O1^i^	0.93	2.45	3.311 (3)	154
C23—H23*B*···O2^i^	0.97	2.53	3.372 (3)	145
C24—H24*B*···O1^i^	0.97	2.31	3.209 (3)	153

Symmetry code: (i) −*x*, *y*−1/2, −*z*+1/2.
